# Evaluation of Resistance Dynamics in Pseudomonas aeruginosa Isolated From ICUs of a Tertiary-Level Hospital in Eastern India: A Five-Year Study

**DOI:** 10.7759/cureus.103639

**Published:** 2026-02-15

**Authors:** B. Prince, A. Raj Kumar Patro, Ipsa Mohapatra, Nipa Singh, Subhra Snigdha Panda, Sujit Pradhan, Nirmala Poddar, Pragati Panda, A. Susanna, Rajesh K Dash, Basanti Kumari Pathi, Dipti Pattnaik

**Affiliations:** 1 Department of Microbiology, Kalinga Institute of Medical Sciences, Bhubaneswar, IND; 2 Department of Community Medicine, Kalinga Institute of Medical Sciences, Bhubaneswar, IND; 3 Department of Critical Care Medicine, Kalinga Institute of Medical Sciences, Bhubaneswar, IND

**Keywords:** amr, ast, icu, mdr, prevalence study, pseudomonas aeruginosa, risk factors, tertiary care hospital

## Abstract

Background

*Pseudomonas aeruginosa*, an important nosocomial bacterial pathogen, poses serious healthcare challenges, particularly in ICUs, leading to morbidity and mortality. Moreover, the emergence of multidrug-resistant (MDR), difficult-to-treat resistant (DTR), and extensive drug-resistant (XDR) isolates has resulted in very limited treatment options. Limited data are available on the antibiotic susceptibility patterns of *P. aeruginosa *in eastern India during the study period. This study aimed to generate insights into the resistance profile of *P. aeruginosa* over a five-year period.

Materials and methods

This retrospective study was conducted from 2021 to 2025 in a tertiary care hospital in Bhubaneswar, India. Samples from ICU patients, including respiratory samples, blood, pus, urine, tissue, and swabs, were cultured on appropriate media. Bacterial identification and antimicrobial susceptibility testing (AST) were performed using the VITEK 2 Compact system (bioMérieux, Marcy-l’Étoile, France). Results were interpreted according to the Clinical & Laboratory Standards Institute (CLSI) 2025 guidelines. All data, including occurrence, antibiogram, comorbidities, and patient predisposing factors, were retrieved from the hospital’s laboratory information system.

Results

A total of 988 *P. aeruginosa* isolates were identified, representing a prevalence rate of 12.08% (988/8174). The highest occurrence was observed in adult ICUs (96.26%, 951/988), followed by pediatric ICUs (3.24%, 32/988) and neonatal ICUs (0.5%, 5/988). Prevalence was higher in males (67%, 659/988) than in females (33%, 329/988). Overall AST patterns revealed the highest susceptibility to amikacin (56%, 523/942), followed by cefepime (53%, 471/891). Resistance was highest for piperacillin/tazobactam (53%, 473/891), followed by imipenem (51%, 446/884) and meropenem (50%, 467/943). Trend analysis showed a decline in resistance to piperacillin/tazobactam, meropenem, imipenem, ciprofloxacin, and levofloxacin over the 2021-2025 period.

Conclusions

This study demonstrates the presence of MDR, non-MDR, DTR, and XDR strains of *P. aeruginosa* in ICUs, with periodic fluctuations in prevalence. This is concerning, as ICU patients are critically ill and infections caused by drug-resistant pathogens may have severe consequences. Continuous antimicrobial stewardship, strict infection control practices, and active surveillance are essential to prevent further escalation of resistance.

## Introduction

In the current era, where antimicrobials are widely available over the counter and extensively used in healthcare, industry, and agriculture, antimicrobial resistance (AMR) has emerged as a serious global crisis. These infections are difficult to treat and often lead to prolonged hospitalization, increased medical costs, significant morbidity, and mortality. In response to the rising burden of AMR, WHO launched the Global Antimicrobial Resistance and Use Surveillance System in 2015 to actively monitor AMR across countries and strengthen findings through systematic data management [1]. In 2019, the United Nations Interagency Coordination Group on AMR reported that drug-resistant infections account for nearly 700,000 deaths annually worldwide, a figure projected to reach approximately 10 million deaths per year by 2050 [2].

AMR can directly or indirectly affect the Sustainable Development Goals (SDGs), which are designed to address global health challenges. Antibiotics are the backbone of therapeutics, and reporting critical trends in AMR is essential for achieving SDG 3, which aims to ensure health and well-being across all age groups [3]. A collaborative global One Health approach has been adopted by quadripartite organizations, including the Food and Agriculture Organization, World Organisation for Animal Health, WHO, and United Nations Environment Programme, to harmonize and improve the health of people, animals, and ecosystems collectively [4]. The Government of India has also launched a nationwide One Health Mission, managed by the Principal Scientific Advisor in coordination with multiple ministries and implemented by the Indian Council of Medical Research (ICMR), to improve health outcomes and address challenges across sectors [5].

*Pseudomonas aeruginosa* is a significant nosocomial pathogen, particularly in ICUs, due to its broad resistance to multiple antibiotic classes, resulting in multidrug-resistant (MDR) and difficult-to-treat resistant (DTR) isolates [6]. MDR isolates are defined as those not susceptible to at least one drug in ≥3 antimicrobial classes, whereas DTR isolates are those not susceptible to ceftazidime, cefepime, ciprofloxacin, imipenem, meropenem, and piperacillin-tazobactam [7]. Extensive drug-resistant (XDR) isolates are defined as those not susceptible to all but ≤2 antimicrobial classes [8].

Antibiotic resistance in *P. aeruginosa *arises from both intrinsic and acquired mechanisms. Intrinsic resistance includes increased AmpC beta-lactamase production, overexpression of efflux pump genes, and downregulation of outer membrane porins. Acquired resistance, such as mutational drug resistance and acquisition of carbapenem resistance genes via horizontal gene transfer, contributes to increased biofilm formation and resistance to last-resort drugs like carbapenems [9]. Genetic alterations in the *mucA22 *allele result in overproduction of alginate, an exopolysaccharide, enhancing biofilm formation in mucoid strains, which are commonly associated with cystic fibrosis infections [10]. Metallo-beta-lactamases such as *blaNDM*, *blaVIM*, and *blaIMP*, and serine carbapenemases like *blaKPC *and *blaOXA*, are primarily responsible for carbapenem resistance [11,12].

Carbapenem-resistant *P. aeruginosa* (CRPA) has been classified as a “high-priority pathogen” on the WHO Bacterial Priority Pathogens List, 2024 [13]. According to the ICMR’s annual report on the Antimicrobial Resistance Research and Surveillance Network 2024, *P. aeruginosa* exhibits higher resistance levels in ICUs compared with wards and OPDs [14].

Due to limited data on the prevalence and antibiogram of* P. aeruginosa *in eastern India during this period, this study aims to provide a detailed analysis of the resistance dynamics of all* P. aeruginosa* isolates, including drug-resistant strains, collected from ICU patients in a tertiary healthcare center in eastern India over a five-year period.

## Materials and methods

Study setting, study duration, and data gathering

This retrospective study was conducted at a 2,600-bed multispecialty hospital in Bhubaneswar, India, from 2021 to 2025, following approval from the Institutional Ethics Committee (approval KIIT/KIMS/IEC/2389/2025). Data on the isolation and antibiogram of *P. aeruginosa* from all ICUs were retrieved from the hospital’s laboratory information system over the five-year period. Patient demographics, comorbidities, and risk factors were extracted from hospital records. To avoid duplication, only the first isolate from each patient was included in the analysis.

Isolates not susceptible to at least one drug from ≥3 antimicrobial classes were defined as MDR [7]. Isolates not susceptible to ceftazidime, cefepime, ciprofloxacin, imipenem, meropenem, and piperacillin-tazobactam were classified as DTR [7]. XDR isolates were defined as those not susceptible to all but ≤2 antimicrobial classes [8].

Sample collection and processing

As part of routine clinical care, specimens, including blood, pus, urine, and swabs, were collected under sterile conditions from ICU patients and transported immediately to the laboratory for bacterial identification and antimicrobial susceptibility testing (AST). All samples were initially screened macroscopically and microscopically to assess cellular components and detect bacterial pathogens using Gram stain.

Blood samples were collected in age-appropriate blood culture bottles and incubated in an automated blood culture system (BacT/ALERT 3D; bioMérieux, Marcy-l’Étoile, France) for up to five days or until flagged positive. Subcultures were performed upon a positive signal. Blood, pus, and other body fluids were inoculated onto blood agar and MacConkey agar (HiMedia Laboratories Pvt. Ltd., Thane, India) and incubated aerobically at 37°C for 24-48 hours. Other specimens were processed according to established guidelines [15,16]. Lactose-nonfermenting, oxidase-positive colonies were further identified using the VITEK 2 ID and AST system.

Bacterial Identification and AST using the VITEK 2 automated system

Isolated colonies were suspended in 0.9% normal saline to prepare a homogeneous 0.5 McFarland solution. Density was verified using VITEK^®^ DENSICHEK^® ^(bioMérieux). Bacterial identification and AST were performed using the VITEK 2 Compact system (bioMérieux) with specific ID and AST panels. The antibiotic panel included piperacillin/tazobactam, ceftazidime, aztreonam, imipenem, meropenem, amikacin, ciprofloxacin, and levofloxacin. Results were interpreted as susceptible, intermediate, or resistant according to the Clinical & Laboratory Standards Institute (CLSI) 2025 guidelines [17]. Intermediate results were analyzed as a separate category from susceptible and resistant.

Statistical analysis

Statistical analyses were performed using R software [18]. Associations between gender, comorbidities, risk factors, and patient outcomes with MDR versus non-MDR isolates were assessed using the chi-square test. Trends in resistance percentages over the years were evaluated using Cochran’s Armitage trend test. P-values were adjusted using the Holm-Bonferroni step-down procedure to reduce Type I error from multiple comparisons. A p-value <0.05 was considered statistically significant. AST patterns were visualized using chord diagrams in R [18], and resistance trends (percentages from 2021-2025) were depicted as heatmaps generated in Python [19].

## Results

**Figure 1:**
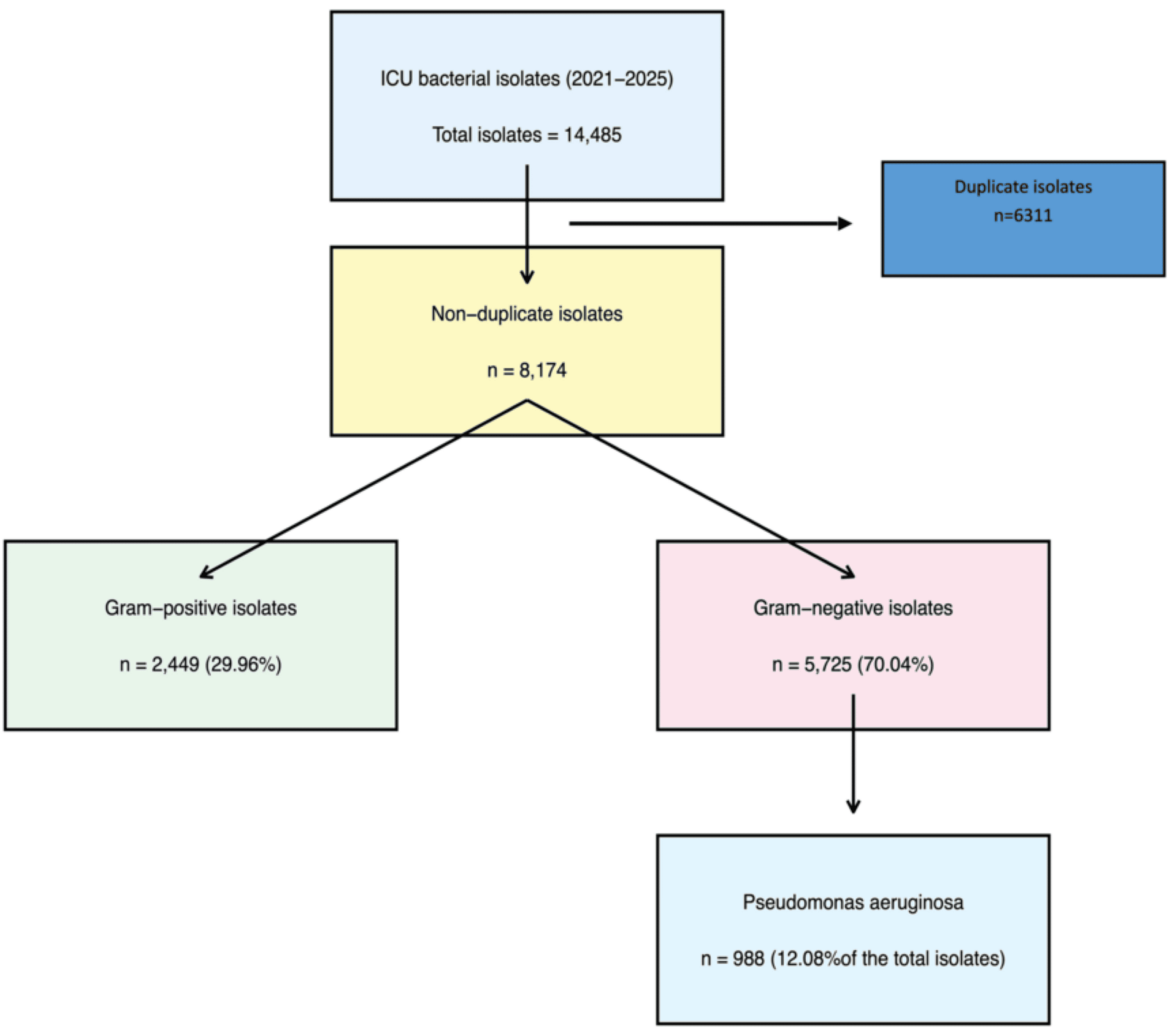
Flow chart depicting the prevalence of Pseudomonas aeruginosa

A total of 14,485 bacterial pathogens were isolated from ICUs during 2021-2025, of which data from 8,174 (56.4%) non-duplicate isolates were retrieved for analysis. Among these non-duplicate isolates, 2,449 (29.96%) were Gram-positive, and 5,725 (70.04%) were Gram-negative. Of the Gram-negative isolates, 988 (12.08%; 95% CI: 11.4-12.8) were identified as *P. aeruginosa* (Figure 1).

**Figure 2:**
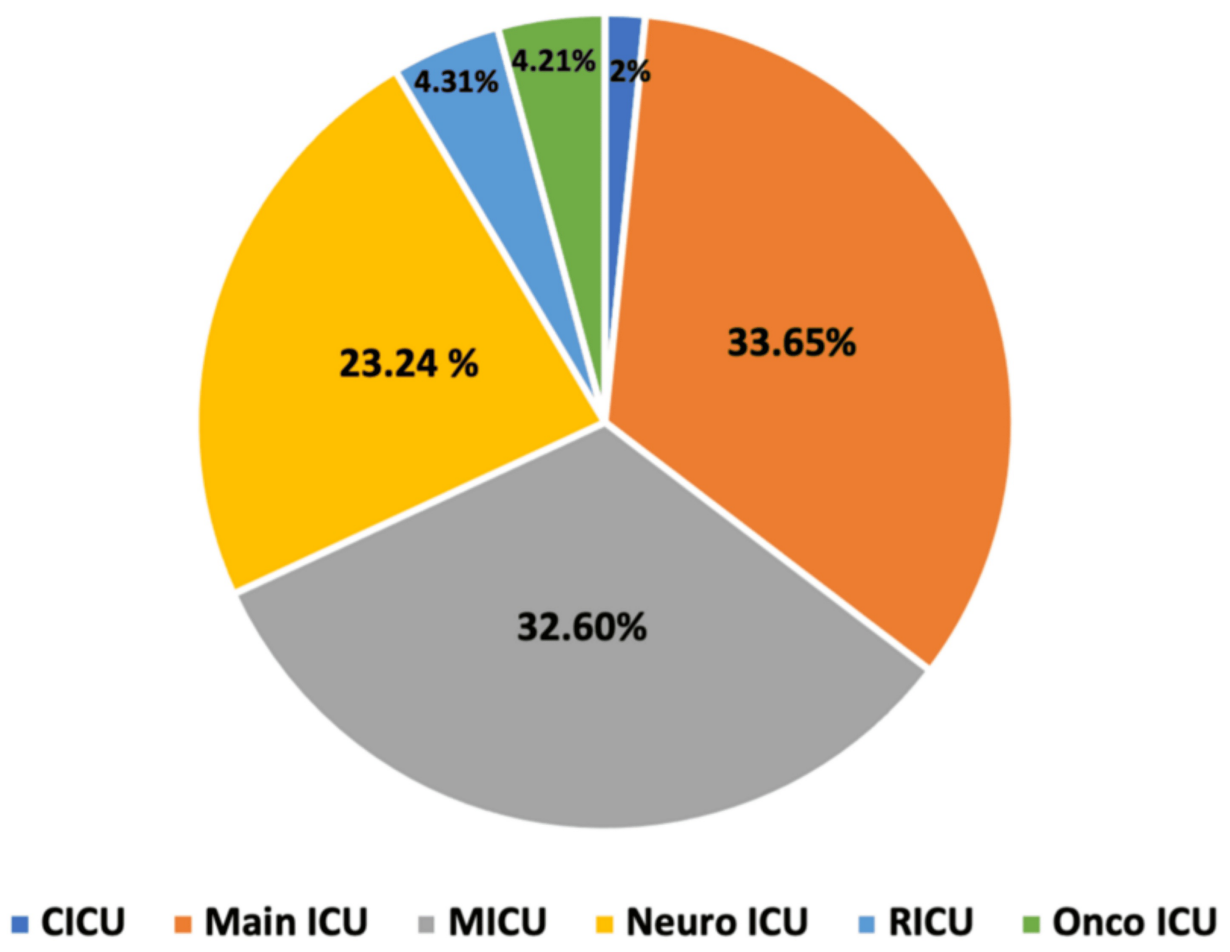
ICU-wise distribution of Pseudomonas aeruginosa CICU, cardiac ICU; MICU, medical ICU; Neuro ICU, neurology ICU; Onco ICU, oncology ICU; RICU, respiratory ICU

The highest occurrence was observed in adult ICUs (96.26%; 951/988), followed by the pediatric ICU (3.24%; 32/988) and neonatal ICU (0.5%; 5/988). Among adult ICU isolates (n = 951), 320 (33.65%) originated from the main ICU, 310 (32.60%) from the medical ICU, 221 (23.24%) from the neuro ICU, 41 (4.31%) from the respiratory ICU, 40 (4.21%) from the oncology ICU, and 19 (2%) from the cardiac ICU (Figure 2).

The majority of isolates were from male patients (67%; 659/988), compared to females (33%; 329/988). Patients were stratified into age groups: 0-10, 11-20, 21-30, 31-40, 41-50, 51-60, and >60 years. The mean age of the study population was 55 ± 17.52 years. Most isolates (436; 44.14%) were obtained from the geriatric cohort (>60 years) (Table 1).

**Table 1:** Demographic details of the patients

Demographic variables	n = 988	Percentage (%)
Gender		
Male	659	67
Female	329	33
Age (in years)		
0-10	27	3
11-20	33	3
21-30	59	6
31-40	87	9
41-50	129	13
51-60	217	22
>60	436	44

The highest proportion of isolates was obtained from respiratory samples (50.10%; 495/988), followed by urine (14.68%; 145/988) and blood samples (14.27%; 141/988).

**Figure 3:**
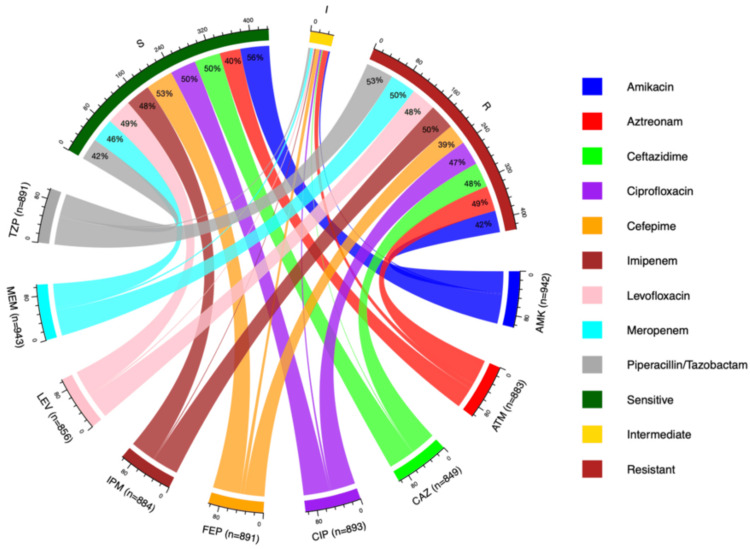
AST pattern of Pseudomonas aeruginosa over five years Susceptible and resistant rates are shown as percentages and interpreted according to the CLSI 2025 guidelines. AMK, amikacin; AST, antimicrobial susceptibility testing; ATM, aztreonam; CAZ, ceftazidime; CIP, ciprofloxacin; CLSI, Clinical & Laboratory Standards Institute; FEP, cefepime; IPM, imipenem; LEV, levofloxacin; MEM, meropenem; TZP, piperacillin/tazobactam

Figure 3 depicts the AST pattern of *P. aeruginosa* over the five-year period from 2021 to 2025. Results were interpreted according to the CLSI 2025 guidelines [17]. Maximum susceptibility was observed for amikacin (56%; 523/942), cefepime (53%; 471/891), ciprofloxacin (50%; 443/893), and ceftazidime (50%; 423/849). In terms of resistance, the highest rates were observed for piperacillin/tazobactam (53%; 473/891), followed by imipenem (51%; 446/884) and meropenem (50%; 467/943).

**Figure 4:**
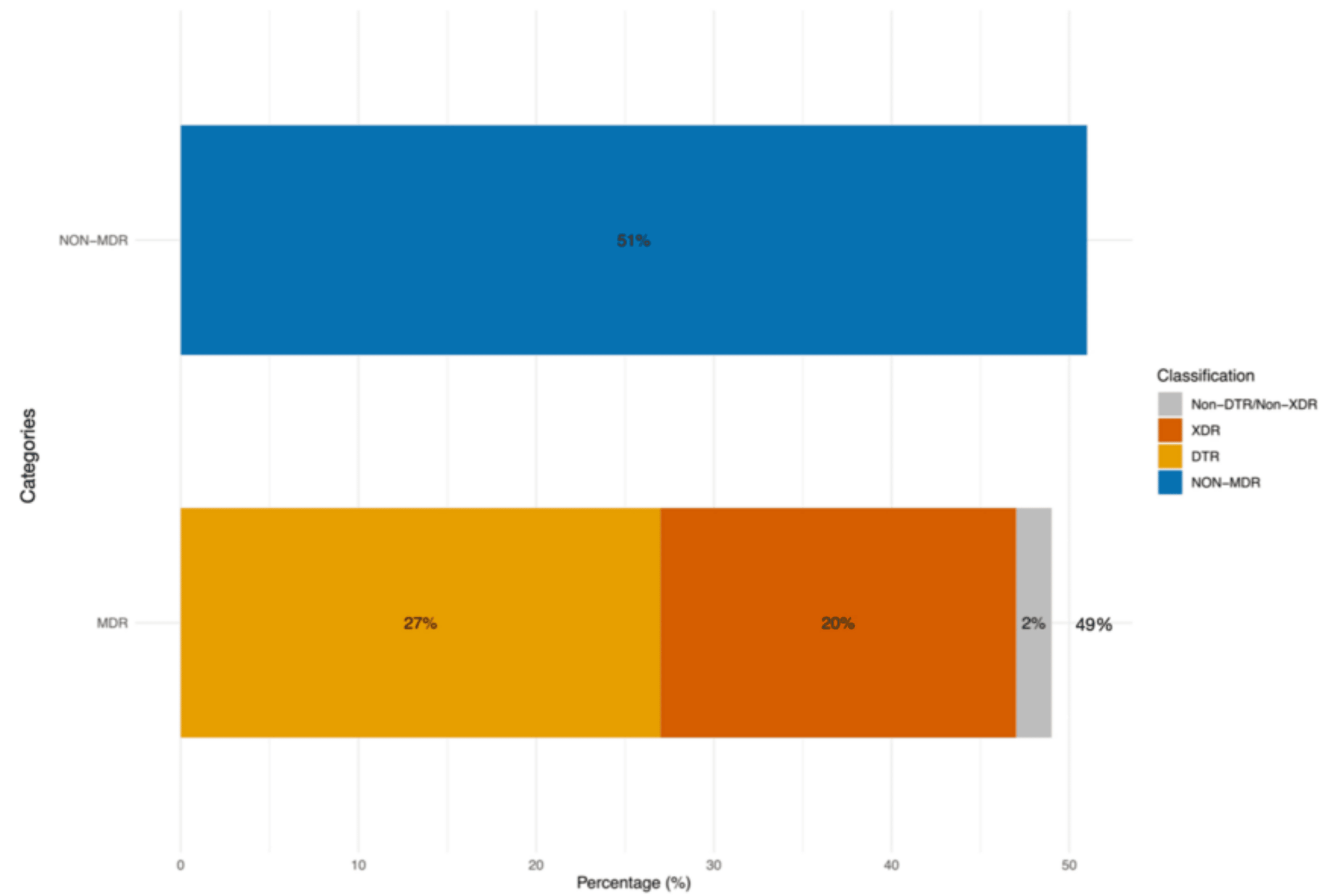
Prevalence of MDR, non-MDR, DTR, and XDR isolates over 2021-2025 (n = 988) DTR, difficult-to-treat; MDR, multidrug-resistant; XDR, extensive drug-resistant

Of the total isolates, 49% (486/988) were identified as MDR, 51% (502/988) as non-MDR, whereas 27% (269/988) and 20% (200/988) were DTR and XDR organisms, respectively (Figure 4).

**Figure 5:**
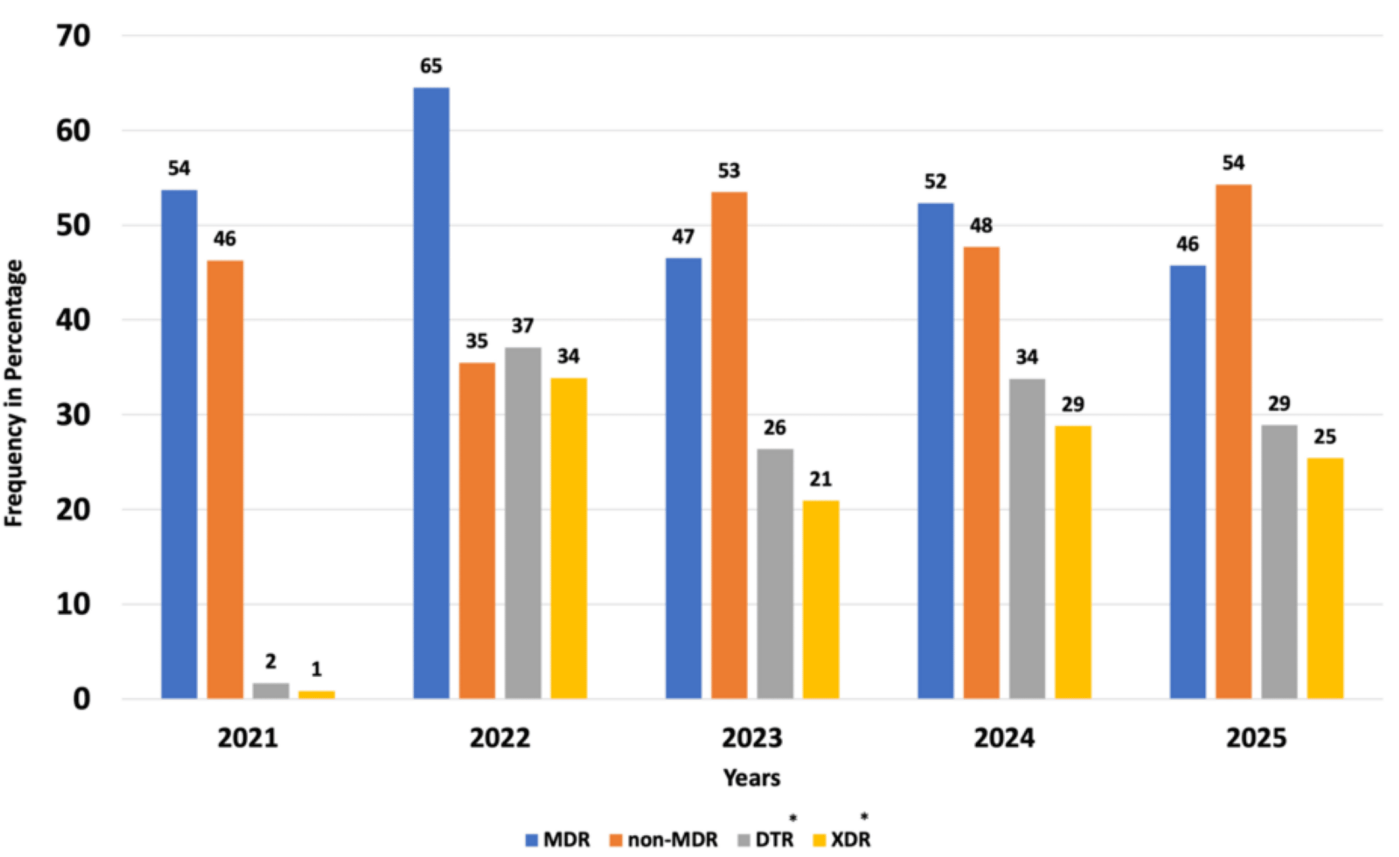
Trends of MDR, non-MDR, DTR, and XDR isolates over five years (percentages) Cochran’s Armitage trend test was used for statistical analysis. Significant trends are indicated as ^*^adjusted p < 0.001. DTR, difficult-to-treat; MDR, multidrug-resistant; XDR, extensive drug-resistant

Figure 5 illustrates the trends over the study period: MDR decreased from 54% (65/121) to 46% (171/374) (adjusted p = 0.0776), while non-MDR increased from 46% (56/121) to 54% (203/374) (adjusted p = 0.0776). DTR and XDR strains also exhibited periodic increases and decreases over the five years (adjusted p < 0.001).

Table 2 presents the adjusted p-values after Holm-Bonferroni correction for multiple trend tests. To ensure monotonicity, the p-value for the non-MDR group was adjusted so that no later p-value was smaller than a previous one.

**Table 2:** Adjusted p-values of trend statistical analysis for MDR and non-MDR categories DTR, difficult-to-treat; MDR, multidrug-resistant; XDR, extensive drug-resistant

Category	Test statistic	df	p-Value	Adjusted p-value
MDR	4.27	1	0.038	0.077
Non-MDR	4.03	1	0.044	0.077
DTR	19.705	1	9.037 × 10⁻⁵	1.26 × 10⁻⁵
XDR	21.719	1	1.26 × 10⁻⁵	2.71 × 10⁻⁵

**Figure 6:**
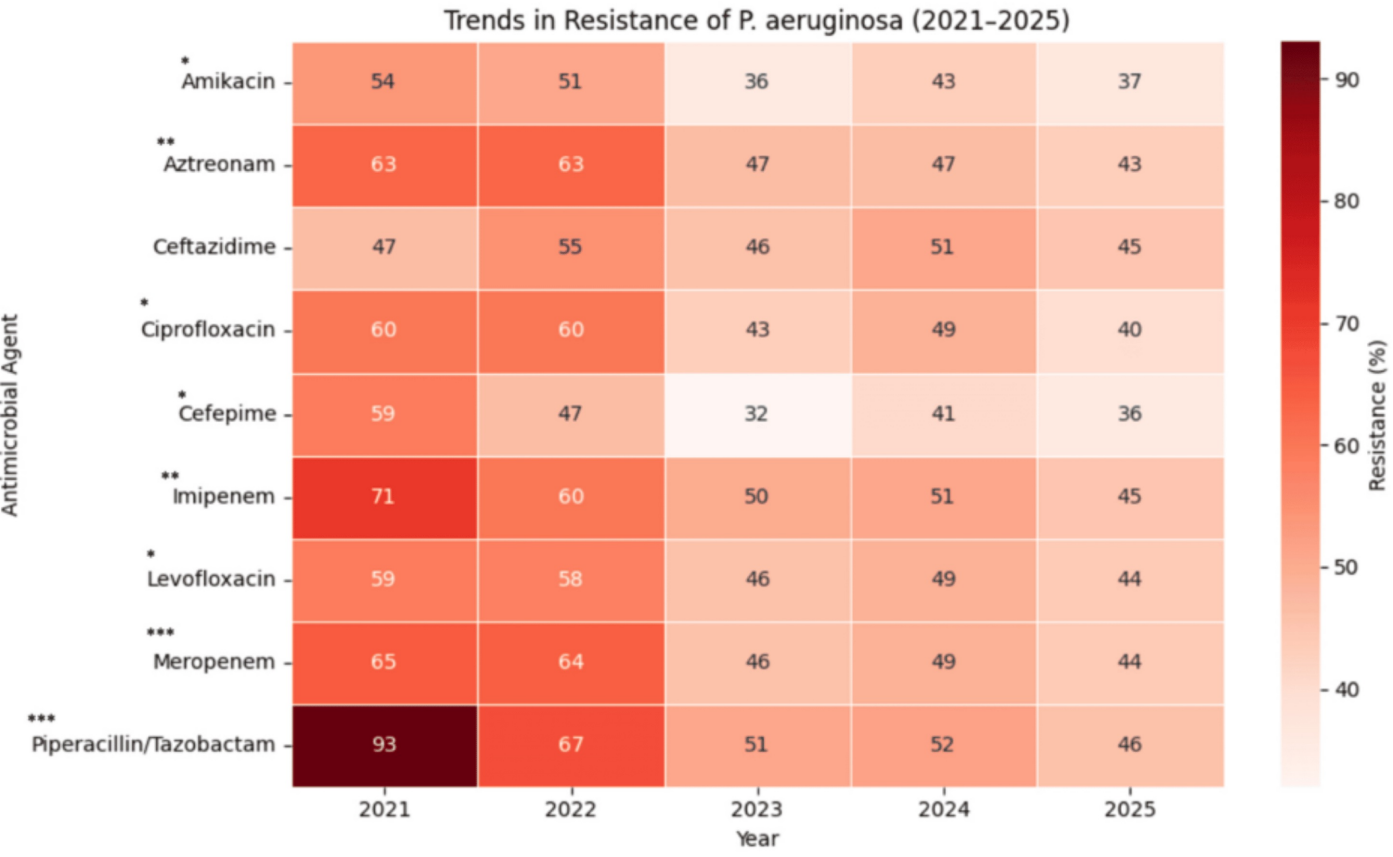
Trends in resistance patterns (percentages) of Pseudomonas aeruginosa over five years Cochran’s Armitage trend test was used to assess temporal trends. ^*^ adjusted p < 0.05; ^** ^adjusted p < 0.01; ^*** ^adjusted p < 0.001

After applying the Holm-Bonferroni correction to all nine trends, a highly significant decreasing trend in AMR from 2021 to 2025 was observed for piperacillin/tazobactam (adjusted p < 0.001) and meropenem (adjusted p < 0.001). Additionally, significant decreasing trends were observed for imipenem (adjusted p < 0.01), aztreonam (adjusted p < 0.01), amikacin (adjusted p < 0.05), ciprofloxacin (adjusted p < 0.05), cefepime (adjusted p < 0.05), and levofloxacin (adjusted p < 0.05). No significant trend was observed for ceftazidime (adjusted p > 0.05) (Figure 6).

Table 3 presents the adjusted p-values of antibiotic resistance trends using the Holm-Bonferroni step-down correction.

**Table 3:** Adjusted p-values of trend statistical analysis for antibiotic categories

Antibiotic	Test statistic	df	p-Value	Adjusted p-value
Amikacin	8.719	1	0.003	0.012
Aztreonam	14.331	1	0.006	0.001
Ceftazidime	0.925	1	0.336	0.336
Ciprofloxacin	9.189	1	0.002	0.012
Cefepime	6.655	1	0.01	0.028
Imipenem	14.295	1	0.0002	0.001
Levofloxacin	5.312	1	0.021	0.042
Meropenem	16.056	1	6.15 × 10⁻⁵	0.0005
Piperacillin/tazobactam	39.217	1	3.79 × 10⁻¹⁰	3.41 × 10⁻⁹

Table 4 shows the association of gender, comorbidities, risk factors, and patient outcomes with MDR status. No significant associations were observed for gender (p = 0.16), diabetes (p = 0.26), hypertension (p = 0.18), alcohol consumption (p = 0.76), smoking (p = 1.00), or death (p = 0.68) with MDR. The chi-square test was used for statistical analysis of these variables.

**Table 4:** Association of gender, comorbidities, risk factors, and outcomes with MDR and non-MDR ^*^ Chi-square test value df, degrees of freedom; HTN, hypertension; MDR, multidrug-resistant

Variables	Total (n = 988)	MDR (n = 486)	Non-MDR (n = 502)	Test statistic	df	p-Value
Gender						
Male	659 (67%)	335 (51%)	324 (49%)	1.95^*^	1	0.16
Female	329 (33%)	151 (46%)	178 (54%)			
Comorbidities						
Diabetic	370 (37%)	173 (47%)	197 (53%)	1.25^*^	1	0.26
Nondiabetic	618 (63%)	313 (51%)	305 (49%)			
HTN	480 (49%)	225 (47%)	255 (53%)	1.83^*^	1	0.18
Non-HTN	508 (51%)	261 (51%)	247 (49%)			
Lifestyle behavior						
Alcoholic	20 (2%)	11 (55%)	9 (45%)	0.09^*^	1	0.76
Nonalcoholic	968 (98%)	475 (49%)	493 (51%)			
Smoker	11 (1%)	6 (55%)	5 (45%)	0^*^	1	1
Nonsmoker	977 (99%)	480 (49%)	497 (51%)			
Outcome						
Death	361 (37%)	174 (48%)	187 (52%)	0.17^*^	1	0.68
Normal discharge	627 (63%)	312 (50%)	315 (50%)			

## Discussion

*P. aeruginosa *is a member of the ESKAPE pathogens, namely *Enterococcus faecium*, *Staphylococcus aureus*, *Klebsiella pneumoniae*, *Acinetobacter baumannii*, *P. aeruginosa*, and *Enterobacter *species, significantly associated with nosocomial infections. Critically ill patients in ICUs are particularly vulnerable to healthcare-associated infections due to multiple comorbidities and compromised immune status. Additionally, the multiple resistance mechanisms and remarkable biofilm-forming ability of *P. aeruginosa *allow severe, persistent infections in susceptible patients. A recent study reported the presence of a novel pap 1 polyesterase in a clinical isolate capable of degrading medical-grade plastics, polycaprolactone, exacerbating the risk in hospital settings [20]. The emergence of CRPA has created numerous therapeutic challenges. Moreover, the worldwide dissemination of drug-resistant strains jeopardizes current treatment regimens, leading to detrimental effects on patient outcomes [11].

In our study, the prevalence of* P. aeruginosa *in ICU settings was 12.08%, which aligns with the findings of Patil et al. (2022) [21], who reported a slightly higher prevalence of 13.66% in ICUs. This study highlights the geriatric cohort (>60 years) as the most vulnerable group, as the majority of isolates were obtained from this population. However, Harsh et al. (2024) [22] documented higher occurrence in the 41-60 age group. These variations may reflect differences in study sites, populations, and time frames.

This study also shows a higher incidence of infection in males compared to females, consistent with Harsh et al. (2024) [22], Venkatasubramanyam et al. (2024) [23], and Thomsen et al. (2023) [24], suggesting that males may be at higher risk. Respiratory samples were the predominant specimen type, indicating a potential risk for conditions such as ventilator-associated pneumonia and chronic obstructive pulmonary disease. In contrast, Bekele et al. (2015) [25] reported a higher incidence of *P. aeruginosa* in females from urine samples. Therefore, the rate of isolation may depend on specimen type, comorbidities, and patient risk factors.

In this study, 49% of isolates were MDR, 51% non-MDR, 27% DTR, and 20% XDR. Selvam et al. (2025) [26] and Gill et al. (2016) [27] reported 52% and 50% MDR *P. aeruginosa*, respectively. Eid et al. (2025) [28] reported 21% DTR isolates, indicating the global emergence of DTR strains. Naik et al. (2021) [29] reported 19.5% XDR *P. aeruginosa *from ocular infections in southern India, suggesting similar prevalence across regions. A decline in MDR rates from 54% to 46% was observed in our study, consistent with Butscheid et al. (2025) [30], who reported reduced resistance post-COVID-19. This decrease may reflect active surveillance, robust antimicrobial stewardship, and infection control measures.

Year-wise analysis of DTR and XDR isolates revealed heterogeneous trends. In 2021, DTR and XDR prevalence was lower than in 2022, followed by a decline from 2022 to 2023. A subsequent rise was observed from 2023 to 2024, with a slight decline from 2024 to 2025. These variations likely reflect antibiotic usage patterns, selective pressure, and the evolution of AMR. Shabi et al. (2025) [31] similarly reported heterogeneous trends in DTR and XDR isolates from 2013 to 2022. Differences may also be influenced by COVID-19, hospital admission rates, variable antibiotic use, and regional resistance patterns.

The antibiogram revealed varying resistance across antibiotic classes. Piperacillin/tazobactam resistance was 53% (n = 891), comparable to Venkatasubramanyam et al. (2024) [23], who reported 32% (n = 562), whereas Vara et al. (2024) [32] reported 14% (n = 140) in ICU settings. Imipenem and meropenem resistance were both 50% (n = 884 and n = 943, respectively), consistent with reports by Vara et al. (2024) [32].

This study also demonstrates a decreasing trend in resistance to piperacillin/tazobactam, imipenem, cefepime, meropenem, ciprofloxacin, aztreonam, levofloxacin, and ceftazidime over five years, potentially reflecting judicious antibiotic use. These findings align with Rana et al. (2025) [33], who reported declining resistance to multiple antibiotics from 2023 to 2024, suggesting that this trend is not confined to ICUs but is also seen in wards, OPDs, and emergency rooms.

This study has several limitations. Being single-center, further multicentric studies with larger sample sizes are needed to better understand *P. aeruginosa *prevalence and resistance trends. The retrospective design limited the assessment of associations between gender, comorbidities, and risk factors. Additionally, results are based solely on phenotypic characterization via conventional and automated systems; molecular analysis of resistance mechanisms was not performed, limiting the depth of interpretation.

## Conclusions

Despite the persistence of overall AMR, trend analysis revealed a decreasing pattern in our ICUs. This underscores the importance of time-trend analyses for effective antimicrobial surveillance, as pooled data may obscure recent improvements. Although the prevalence of MDR strains decreased slightly during the study period, MDR, DTR, and XDR strains continue to be reported in ICUs, which remains a significant concern. Non-MDR* P. aeruginosa *isolates continue to show an increasing trend in our setting. *P. aeruginosa* remains a critical nosocomial pathogen in ICU settings, where critically ill patients are highly vulnerable to severe infections. Therefore, continued active AMR surveillance, training of healthcare staff, robust infection prevention and control strategies, and rational antibiotic stewardship are essential to limit the persistence of resistant strains in ICUs.
